# Dissecting the Role of PCDH19 in Clustering Epilepsy by Exploiting Patient-Specific Models of Neurogenesis

**DOI:** 10.3390/jcm10132754

**Published:** 2021-06-23

**Authors:** Rossella Borghi, Valentina Magliocca, Stefania Petrini, Libenzio Adrian Conti, Sandra Moreno, Enrico Bertini, Marco Tartaglia, Claudia Compagnucci

**Affiliations:** 1 Genetics and Rare Diseases Research Division, Bambino Gesù Children’s Hospital, IRCCS, 00146 Rome, Italy; rossella.borghi@opbg.net (R.B.); valentina.magliocca@opbg.net (V.M.); enricosilvio.bertini@opbg.net (E.B.); marco.tartaglia@opbg.net (M.T.); 2 Department of Science, University “Roma Tre”, 00146 Rome, Italy; sandra.moreno@uniroma3.it; 3 Confocal Microscopy Core Facility, Research Laboratories, Bambino Gesù Children’s Hospital, IRCCS, 00146 Rome, Italy; stefania.petrini@opbg.net (S.P.); libenzioadrian.conti@opbg.net (L.A.C.)

**Keywords:** iPSCs, neurons, PCDH19, neuronal progenitor cells, neurogenesis, disease modeling, neurological disease

## Abstract

PCDH19-related epilepsy is a rare genetic disease caused by defective function of PCDH19, a calcium-dependent cell–cell adhesion protein of the cadherin superfamily. This disorder is characterized by a heterogeneous phenotypic spectrum, with partial and generalized febrile convulsions that are gradually increasing in frequency. Developmental regression may occur during disease progression. Patients may present with intellectual disability (ID), behavioral problems, motor and language delay, and a low motor tone. In most cases, seizures are resistant to treatment, but their frequency decreases with age, and some patients may even become seizure-free. ID generally persists after seizure remission, making neurological abnormalities the main clinical issue in affected individuals. An effective treatment is lacking. In vitro studies using patient-derived induced pluripotent stem cells (iPSCs) reported accelerated neural differentiation as a major endophenotype associated with PCDH19 mutations. By using this in vitro model system, we show that accelerated in vitro neurogenesis is associated with a defect in the cell division plane at the neural progenitors stage. We also provide evidence that altered PCDH19 function affects proper mitotic spindle orientation. Our findings identify an altered equilibrium between symmetric versus asymmetric cell division as a previously unrecognized mechanism contributing to the pathogenesis of this rare epileptic encephalopathy.

## 1. Introduction

PCDH19-related epilepsy (MIM 300088) is a rare, genetic, drug-resistant developmental and epileptic encephalopathy, generally affecting females, and characterized by early-onset intractable seizures (9 months, on average). The disorder, which was first reported fifty years ago by Juberg and Hellman [1], represents one of the most diffuse monogenetic epileptic forms in the pediatric population [2,3,4,5], and recent molecular epidemiologic studies indicate PCDH19 as the second most clinically relevant gene in epilepsy after *SCN1A* [6,7,8,9]. The disease is characterized by a wide phenotypic spectrum, ranging from benign focal epilepsy with normal cognitive function to severe generalized or multifocal epilepsy resembling Dravet syndrome, with a more favorable prognosis [6,10,11].

PCDH19 is localized on the long arm of chromosome X (Xq22.3) and encodes a membrane calcium-dependent cell–cell adhesion glycoprotein of the protocadherin family [11,12,13]. PCDH19 is characterized by six extracellular cadherin repeats with conserved calcium binding sequences, a transmembrane domain, and an intracellular region with two conserved motifs (CM1 and CM2) at the C terminus [13]. Like the classical cadherins, the main function of protocadherins is to enhance cell aggregation in a homophilic fashion, although protocadherin-mediated cell–cell adhesion is generally weaker than the classical cadherin-mediated adhesion [14,15]. 

PCDH19 function is still poorly characterized but is known to be required in several developmental processes, including cell migration, axonal outgrowth, and synaptogenesis, which are necessary for proper neurodevelopment [16,17]. It has recently been reported that PCDH19 expression is post-transcriptionally regulated by miR-484, which inhibits its translation [18]. Since miR-484 positively regulates neurogenesis by promoting neuronal progenitor differentiation, it has been hypothesized that PCDH19 plays a role in regulating neuronal progenitor fate [18]. Indeed, it has been shown that PCDH19 expression suppresses the differentiation of neuronal progenitors, while its inhibition reduces the proliferation of radial glia, promoting differentiation into basal progenitors [18]. Studies on a *Pcdh19* KO murine model showed the premature differentiation of neural progenitor cells associated with a reduction in apical–basal polarity, altered migration of cortical neurons, and abnormal cell sorting, suggesting impaired neurogenesis, although morphological analyses did not reveal gross abnormalities in mice brain structures [18,19,20,21,22,23]. Recently, it has been demonstrated that PCDH19 has a calcium-dependent function in cell–cell adhesion, and studies in mice highlighted the role of the protein in determining cell adhesion affinities during cortical development [21]. Moreover, in zebrafish neural stem and progenitor cells, Pcdh19 has been shown to directly interact with N-cadherin (cdh2/ncad), an integral component of adherens junctions, to form a complex with strong adhesive properties and mediate morphogenic movements during brain development [17,24]. Importantly, *Pcdh19* mutant mice, generated using the CRISPR/Cas9 system showed that, in *Pcdh19^HET(female)^* mice, the interactions between Pcdh19 and Ncad proteins are mismatched, thus impairing Ncad-dependent signaling and presynaptic development [25].

PCDH19-related epilepsy is characterized by an unusual pattern of inheritance, as affected patients are generally females heterozygous for a pathogenic PCDH19 variant [6]. Female homozygous for a pathogenic variant does not express signs of the disease; similarly, male hemizygous for a disease-associated variant is clinically unaffected. The PCDH19 gene is in a region subject to chromosome X inactivation, and in heterozygous females, two cellular populations, expressing either the wild-type or mutated allele coexist. According to the “cellular interference” model hypothesized by Depienne and colleagues [6], the coexistence of this mixed population seems to be detrimental to the normal functioning of brain cells. In males, the uniform expression of a pathogenic PCDH19 allele does not affect the proper functioning of neuronal cells. Consistent with this model, mosaic males develop the disease due to the concurrence of two neuronal populations expressing the wild-type or pathogenic PCDH19 allele [3,6,26,27]. Based on these recent findings, the name of this disorder has recently been changed from “female restricted epilepsy with intellectual disability” to PCDH19 clustering epilepsy (PCDH19-CE) [28]. The altered segregation of the two different populations of cells, *Pcdh19*^WT^ and *Pcdh19*^mut^, has been demonstrated in the developing brain of heterozygous female mice [21], but not in hemizygous *Pcdh19* KO male mice [29]. These findings provide experimental evidence of the cellular interference as a key pathogenic mechanism underlying PCDH19-CE. In addition to this, a non-mosaic male with Klinefelter syndrome (47, XXY) was found to be affected by PCDH19-CE [30], thus confirming the “cellular interference” theory and ruling out the possibility of a compensatory rescue in males.

The study of molecular mechanisms of disease has traditionally involved the use of genetically modified animal models, which may fail to recapitulate the critical aspects of disease, especially in neurologic pathologies [31]. A complementary experimental approach to investigating the pathogenetic mechanisms underlying neurodevelopmental disorders is based on the use of patient-derived induced pluripotent stem cells (iPSCs), which allow for the in vitro recapitulation of human neurogenesis [32]. iPSCs represent a particularly informative model to explore the pathomechanisms of diseases in the proper cellular context, which are otherwise experimentally inaccessible. Following the studies of Yamanaka and colleagues [33], the somatic reprogramming of human cells overcame the limitations of dissecting human diseases due to the unavailability of relevant human tissues. Somatic cell reprogramming allows for the derivation of patient-specific iPSCs, which can be differentiated into disease-pertinent cell-types and used as an informative model system before, during and following differentiation.

Here, we generated patients’ fibroblast reprogrammed iPSCs as an in vitro model for PCDH19-CE. Specifically, by modeling cortical neurons derived from iPSCs obtained from a male patient with a postzygotic pathogenic variant in PCDH19, we show that the accelerated differentiation of PCDH19-mutated iPSCs is associated with an altered orientation of cell division. We also provide evidence that cells expressing a mutated PCDH19 allele have an aberrant mitotic spindle.

## 2. Experimental Section

### 2.1. Derivation of iPSCs

The studies were conducted in compliance with the Code of Ethics of the World Medical Association (Declaration of Helsinki), and with national legislation and institutional guidelines (local institutional ethical committee, Ref. 1702_OPBG_2018, date of approval 11 February 2019). PCDH19^mut^ iPSCs were derived from primary skin fibroblasts of an affected male individual (c.1352C>T, p.Pro451Leu; [25]), and control (CTRL) iPSCs were derived from a male, age-matched, healthy individual. An additional CTRL iPSC line was purchased from System Biosciences. All experiments were performed using the two control lines. Since no difference was observed among CTRL lines in all experiments, the figures report only one CTRL iPSC line. 

Cells were reprogrammed in house using non-integrating episomal technology (Minicircle DNA and mc-iPS Cells, Euroclone, Milan, Italy, Cat. SC301A-1), by nucleofection, using a vector containing five reprogramming factors (Oct4, Sox2, Lin28, Klf4, and L-Myc) [34]. Specifically, dermal fibroblasts obtained from the mosaic male patient by skin biopsy were cultured in Dulbecco’s Modified Eagle Medium (DMEM D5671, Sigma Aldrich, St. Louis, MO, USA) supplemented with 10% of Fetal Bovine Serum (FBS) (10082-147, Gibco, Waltham, MA, USA) at 37 °C, 5% CO_2_. At passage 3 (confluence of 75–80%), 1 × 10^5^ fibroblasts were prepared for reprogramming by nucleofection of the episomal vectors using Epi5 Episomal iPSC Reprogramming Kit (A15960, Invitrogen, Waltham, MA, USA) and the Nucleofection kit P2 solution (LOV4XP2024, Lonza, Basilea, Switzerland) with 4D-Nucleofector System (Lonza, Basilea, Switzerland). Transfected cells were plated in 6-well plates pre-coated with Matrigel-Matrix hESC-qualified (#354277, Corning, New York, NY, USA) in DMEM/FBS medium. Twenty-four hours after nucleofection, the medium was replaced with N2B27 medium (composed by: DMEM/F12 with L-Glutamine and 25mM Hepes (ECM0095L, Euroclone, Milan, Italy), N2 supplement (17502-048, Gibco, Waltham, MA, USA), B27 supplement (17504-044, Gibco, Waltham, MA, USA), 100 μM of β-Mercaptoethanol (21985-023, Gibco, Waltham, MA, USA), 0.1 mM MEM Non-Essential Amino Acids Solution (11140-050, Gibco, Waltham, MA, USA) supplemented with 100 ng/mL bFGF. The medium was changed daily until day 15, when N2B27 medium was substituted with Essential 8 medium (A1517001, Gibco, Waltham, MA, USA).

The first iPSCs colonies appeared at around day 18, and the Essential 8 medium was changed with mTeSR1 (85850, STEMCELL Technologies, Vancouver, BC, Canada). iPSCs colonies were picked under a sterile hood with EVOS microscopy system (Thermo Fisher Scientific, Waltham, MA, USA) and transferred into a 24-well plate, pre-coated with Matrigel and cultured in mTeSR1 medium for expansion. The criteria used to select the best cell clones were based on colony morphology (characterized by rounded and sharp colony edges). 

### 2.2. Alkaline Phosphatase Assay

Alkaline phosphatase (ALP) staining was performed, following the manufacturer’s instructions (86R-1, Merck KGaA, Darmstadt, Germany). Cells were incubated at RT for 30 min with a solution based on naphthol AS-BI and fast red violet LB (86R-1KT, Sigma Aldrich, St. Louis, MO, USA). The cells were photographed using a Leica DM1000 (Leica Microsystems, Wetzlar, Germany) equipped with Leica LAS X software (Leica Microsystems, Wetzlar, Germany).

### 2.3. Immunofluorescence Assay for Pluripotency

Immunofluorescence assays were performed using Pluripotent Stem Cell 4-Marker Immunocytochemistry Kit (A24881, Invitrogen, Waltham, MA, USA). Cells were blocked with 5% bovine serum albumin (BSA) (10775835001, Roche, Basilea, Switzerland) and permeabilized with 0.1% Triton X-100 (Sigma Aldrich, St. Louis, MO, USA) for 1 h at RT. Primary antibodies were incubated for 2 h at room temperature (RT) and included anti-OCT4 (1:100, rabbit), anti-SSEA4 (1:250, mouse), anti-SOX2 (1:200, rat), anti-TRA-1-60 (1:100, mouse).

### 2.4. In Vitro Trilineage Differentiation Assay

Pluripotency characterization was also performed using the STEMdiff Trilineage Differentiation Kit (05230; STEMCELL Technologies Inc., Vancouver, BC, Canada) according to manufacturer’s instructions. Briefly, iPSCs were plated onto Matrigel and treated with endoderm or mesoderm differentiation media for 5 days or ectoderm differentiation media for 7 days. Cells were then fixed, stained, and imaged to document their positivity to anti-SOX17 (1:3.200, ON at 4 °C, rabbit, 81778 Cell Signaling, Danvers, MA, USA), anti-NCAM (1:400, On at 4 °C, rabbit, 89861 Cell Signaling, Danvers, MA, USA) and anti-TBXT (1:1.600, ON at 4 °C, rabbit, 81694 Cell Signaling, Danvers, MA, USA).

### 2.5. Genome Integrity Assay

DNA extraction was executed using NucleoSpin Tissue (740952, Macherey-Nagel, Düren, Germany) and following user’s manual. To perform a genetic analysis of the obtained clones of iPSCs, we used a qPCR-based kit (hPSC Genetic Analysis Kit 07550, STEMCELL Technologies, Vancouver, BC, Canada). The assay is able to detect the most common karyotypic abnormalities reported in human iPSCs (Chr 1q, Chr 4p, Chr 8q, Chr 10p, Chr 12p, Chr 17q, Chr 18q, Chr 20q, Chr Xp). The assay uses a double-quenched probe with a 5-carboxyfluorescein (5-FAM) dye, and it was used following manufacturer’s instructions. Data were analyzed with Genetic Analysis Application supplier (STEMCELL Technologies, Vancouver, BC, Canada).

### 2.6. Maintenance of iPSCs

iPSCs were grown in feeder-free condition using Matrigel in mTeSR1. When the iPSCs were 70–80% confluent, they were passaged 1:4 and transferred to new wells and incubated at 37 °C, 5% CO_2_; the medium was changed every day and the cells split every three days. 

### 2.7. Differentiation of iPSCs into Cortical Neurons

Neuronal differentiation protocol was adapted from [35]. iPSCs were plated into a 6-well plate, pre-coated with Matrigel. When cells reached a confluence of 30%, iPSCs medium was replaced with a chemically defined medium containing DMEM/F12 (31331028, Gibco, Waltham, MA, USA), N2 Supplement and 2 μg/mL Heparin (Sigma Aldrich, St. Louis, MO, USA) for 16 days. On day 17, the medium was replaced with Neurobasal Medium (21103049, Gibco), supplemented with N2, B27, BDNF (10 ng/mL, Peprotech, Cranbury, NJ, USA), GDNF (10 ng/mL, Peprotech, Cranbury, NJ, USA) and IGF1 (10 ng/mL, Peprotech, Cranbury, NJ, USA) until day 30.

### 2.8. Brain Organoids Generation, Culturing, and Analysis

Brain organoids were generated from iPSCs using culture mediums for the establishment of human iPSC-derived cerebral organoids (STEMDIFF Cerebral Organoid Kit 08570, STEMCELL Technologies, Vancouver, BC, Canada) based on the formulation published by [36,37]. Cerebral organoid formation was initiated through an intermediate embryoid body (EB) formation step, followed by the induction and expansion of neuroepithelia. iPSCs were seeded at a confluence of 9000 cells/well in a 96-well, ultra-low attachment plate (7007, Corning, New York, NY, USA) in EB formation medium. On the 5th day, the medium was changed with brain organoid induction medium. On the 7th day, each organoid was embedded into a Matrigel dome at the center of the well in a 24-well plate (FALCON), in the brain organoid expansion medium. From the 10th day, the brain organoids were maintained in maturation medium. For imaging, the IncuCyte S3 time-lapse microscopy system (Sartorius) (Essen BioScience, Ann Arbor, MI, USA) was used to image the wells every 6 h. Imaging was performed for 8 days (from the 8th to the 16th day) at 37 °C. Phase images were acquired for every experiment. Analysis parameters for software module-processing definitions were optimized individually for each experiment according to the workflow outlined in the manufacturer’s manual. The optimized processing definitions were subsequently used for image analysis, focusing on the parameter of the Organoid object total area. Microplate graphs were generated using the time plot feature in the graph/export menu of the IncuCyte software. The raw data of organoid growth were exported to Microsoft Excel and GraphPad Prism was used to calculate mean values ± SEM and perform ad hoc statistical analyses.

### 2.9. Immunostaining

For immunocytochemistry, cells were fixed with 4% paraformaldehyde (157-8, Electron microscopy sciences, Fort Washington, Pennsylvania, USA) for 10 min at RT, washed twice with phosphate-buffered saline (PBS) without Ca^2+^ and Mg^2+^. For permeabilization, cells were incubated with blocking solution containing 5% BSA and 0.1% Triton X-100 in PBS for 1 h at RT. Primary antibodies included anti-PCDH19 (1:100, ON at 4 °C, rabbit, HPA001461 Sigma Aldrich, St. Louis, MO, USA), anti-βIII-tubulin (1:500, 2 h at RT, mouse, T8578, Sigma Aldrich, St. Louis, MO, USA), anti-γ-tubulin (1:1000, ON at 4 °C, mouse, sc-51715, Santa Cruz, Dallas, TX, USA), anti-β-tubulin (1:100, ON at 4 °C, rabbit, 2146 Cell Signaling, Danvers, MA, USA), anti-centriolin (1:100, ON at 4 °C, mouse, sc-365521, Santa Cruz, Dallas, TX, USA). Secondary antibodies used were conjugated with either Alexa 488 (anti-mouse: A11017, anti-rabbit: A11070, Thermo Fisher Scientific, Waltham, MA, USA), Alexa 555 (anti-mouse: A21425, anti-rabbit: A21430, Thermo Fisher Scientific, Waltham, MA, USA). Primary antibodies were diluted in blocking solution and incubated for at least 1 h at RT. Prior to and following the 1 h incubation period with the corresponding secondary antibody (Alexa Fluor, 1:500, Thermo Fisher Scientific, Waltham, MA, USA), cells were washed two times in PBS, for 5 min each. Nuclei were counter-stained with Hoechst 33342 (1:10,000, H3570, Merck KGaA, Darmstadt, Germany) and coverslips were mounted using PBS/glycerol (1:1). All images were acquired using the confocal microscope Leica SP8 (Leica Microsystems, Wetzlar, Germany) in combination with the LAS-X software (Leica Microsystems, Wetzlar, Germany). Confocal z-stacks were imported into LAS-X 3D software (Leica Microsystems, Wetzlar, Germany) to obtain their 3D surface rendering. Metaphases count was performed using NDP.view2 Image viewing Software (Hamamatsu Photonics, Hamamatsu City, Shizuoka, Japan) on immunofluorescence for βIII-tubulin. Stained samples were previously acquired for their entire surface with a digital scanner NanoZoomer S60 (Hamamatsu Photonics, Hamamatsu City, Shizuoka, Japan).

### 2.10. Neurite Length Assay

Cortical neurons were plated at a density of 5000 cells/well in a 96-well plate (92696, TPP) pre-coated with Matrigel and neurites’ length was measured using the Incucyte System (Essen BioScience, Ann Arbor, MI, USA) with the Neurite Analysis application for Neurolight labeled cells. The cells were infected with a lentiviral-based vector encoding with an orange fluorescent protein (Incucyte Neurolight Lentivirus 4807, Essen BioScience, Ann Arbor, MI, USA). Well plates were imaged every 2 h in the IncuCyte S3 time-lapse microscopy system (Essen BioScience, Ann Arbor, MI, USA). Imaging was performed for 3 days (from the 10th to the 13th day of neural differentiation) and for 6 days at the end of the differentiation (from the 35th to the 41st day) at 37 °C. Phase-contrast and fluorescent images were acquired for every experiment. Analysis parameters for NeuroTrack software module-processing definitions were optimized individually for each experiment according to the workflow outlined in the manufacturer’s manual. The optimized processing definitions were subsequently used for real-time image analysis. Microplate graphs were generated using the time plot feature in the graph/export menu of the IncuCyte Zoom software (Essen BioScience, Ann Arbor, MI, USA). Raw data neurite lengths were exported to Microsoft Excel and GraphPad Prism to calculate mean values ± SEM and perform ad hoc statistical analyses.

### 2.11. RNA Isolation and Reverse Transcriptase-Polymerase Chain Reaction Analysis

Total RNA was extracted from iPSCs or from iPSCs-derived cortical neurons with the single-step acid phenol method using TRIzol (15596018; Thermo Fisher Scientific, Waltham, MA, USA) according to the manufacturer’s instructions. Each RNA sample was treated with recombinant DNase I (AM2235, Thermo Fisher Scientific, Waltham, MA, USA) and quantified by NanoDrop 2000 (Thermo Fisher Scientific Life Sciences, Waltham, MA, USA). The reverse transcription reaction was performed in 20 µL, starting from 1 µg of total RNA, and cDNA was generated by using the ImProm-II Reverse Transcription System (A3800; Promega, Madison, WI, USA) or Superscript II reverse transcriptase (18064; Thermo Fisher Scientific, Waltham, MA, USA) using random hexamers. Three independent reverse-transcription quantitative real-time PCR (RT-qPCR) were performed for each sample. 

### 2.12. Quantitative Real-Time PCR

Total RNA (0.2–1 µg) from iPSCs or differentiating iPSCs into cortical neurons was used for RT–qPCR using M–MLV reverse transcriptase (Thermo Fisher Scientific, Waltham, MA, USA). Five percent of the reaction was used as template, together with the primers specific to the analyzed list of genes (Table 1). qPCR analysis was performed using Power SYBR Green PCR Master Mix (4367659, Applied Biosystems, Waltham, MA, USA) and the 7900HT Fast Real-Time PCR System (Applied Biosystem, Waltham, MA, USA), according to the manufacturer’s instructions. The ΔΔCt method was used to calculate the fold change in gene expression. ΔCt values were obtained by subtracting the Ct value obtained for the specific gene from the Ct value of the housekeeping gene for each sample and normalized the housekeeping gene levels (TBP). Data represent fold increase versus control sample, calculated by the 2t -(ΔΔC) formula. Expression levels were represented in arbitrary units, calculated as a relative-fold increase compared with the control sample, which was arbitrarily set to 1. Quantitative RT-PCRs were repeated in triplicate from at least two independent experiments. 

### 2.13. PCDH19 mRNA Silencing

To silence PCDH19 a heterogeneous mixture of small interfering RNA (siRNA) was used to obtain highly specific and effective gene silencing with low risk for off-target effects. In particular, 5 × 10^5^ of CTRL-iPSCs were electroporated with 500 ng of endoribonuclease prepared siRNA for human PCDH19 (MISSION esiRNA EHU032881, Sigma Aldrich, St. Louis, MO, USA), using Nucleofection kit P2 solution with 4D-Nucleofector System. Cells were plated in 6-well plates containing cover glasses pre-coated with Matrigel. Twenty-four hours after nucleofection, cover glasses were fixed to perform an immunofluorescence assay with anti-PCDH19 antibody (HPA001461, Sigma Aldrich, St. Louis, MO, USA) and the remaining cells were collected to perform protein extraction for Western blot analyses.

### 2.14. Western Blotting

For Western blot analyses, cells were lysed in RIPA buffer (R0278, Sigma Aldrich, St. Louis, MO, USA), supplemented with complete protease inhibitor cocktail (88668, Thermo Fisher Scientific, Waltham, MA, USA). Proteins were separated by SDS-PAGE and transferred to nitrocellulose membrane. Membranes blocked in 5% milk for 1 h at RT. Primary antibodies were incubated overnight at 4 °C. Secondary antibody–HRP conjugates were incubated for 1 h at RT and membranes stained with Clarity Western ECL Blotting Substrate (170-5060, Biorad, Hercules, CA, USA). The following primary antibodies were used: anti-PORIN (1:10,000, 1 h at RT, mouse, MSA05, Mitoscience, Eugene, OR, USA), anti-PCDH19 (1:1000, ON at 4 °C, rabbit, HPA001461 Sigma Aldrich, St. Louis, MO, USA).

### 2.15. Calcium Imaging

Calcium studies were carried out by plating cells on glass bottom microwell dishes (81156, Ibidi, Martinsried, Planegg, Germany) and incubating them in Tyrode’s solution (in mM: 129 NaCl, 5 KCl, 2 CaCl_2_, 1 MgCl_2_, 25 HEPES, 30 Glucose, pH 7.4) supplemented with 5 mM Fluo-4 (F10489, ThermoFisher Scientific, Waltham, MA, USA) for 15 min at RT and 5% CO_2_ in the dark. Fluorescence microscopy was performed using a Leica SP8X resonant scanner confocal microscope using time-series frames of 2 fs/sec. After baseline interval, Ionomycin (I24222, Thermo Fisher Scientific, Waltham, MA, USA) diluted in Tyrode’s solution was added (adapted from [38]). Following the addition of (20 μM) ionomycin, the maximum peak in fluorescence was recorded and, following 30 s (60 mM) EGTA (SLBR7504V, Sigma Aldrich, St. Louis, MO, USA), was supplemented to the media to measure minimum fluorescence intensity. For each biological replicate, 10–20 cells were measured. Traces in the graphs represent the normalized average fluorescence intensity change over time. For quantification, the area under the curve (AUC) of the whole Fluo-4 fluorescence peak area was determined using GraphPad Prism (San Diego, CA, USA).

### 2.16. Statistical Analyses 

Data were expressed as the mean and standard error of the mean (mean ± SEM), where the normality of the distribution could be verified. For all experiments, multiple technical replicates and biological replicates were utilized (indicating the number of biological replicates with n). Detailed information regarding the number of replicates for each experiment can be found in the respective figure legend. Where *n* = 3, we assessed that the statistical test could be applied, and the distribution of the data could be verified. Significance was assessed using parametric tests (Student’s *t* test, ANOVA) for normally distributed data and non-parametric tests (Mann–Whitney U test, Kruskal–Wallis) when normal distribution could not be verified. A *p* value < 0.05 was considered to indicate significance. Data were analyzed using GraphPad Prism software (Prism 8.0.2, GraphPad Software, San Diego, CA, USA) and Microsoft Excel (Microsoft, Redmond, Washington, DC, USA).

## 3. Results

### 3.1. Derivation of PCDH19-CE iPSCs 

**Figure 1:**
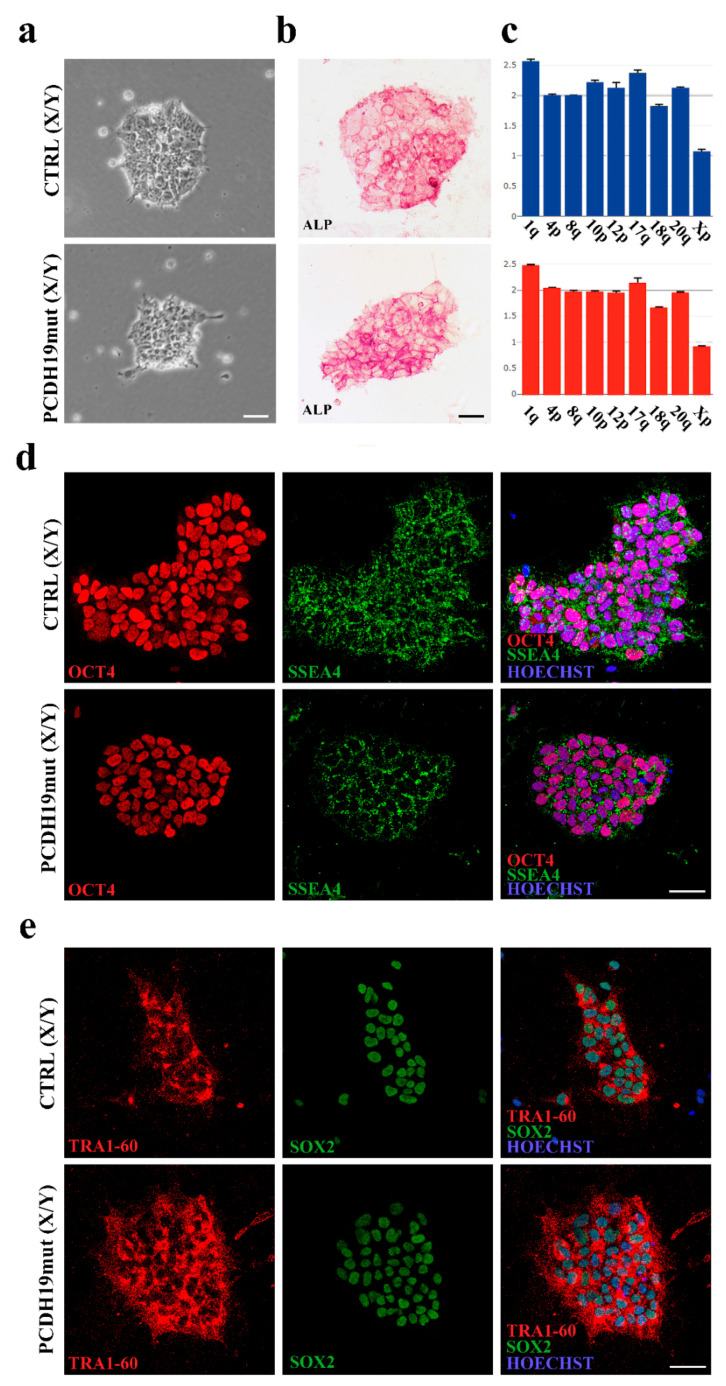
Characterization of iPSCs obtained from fibroblasts of a mosaic male patient (PCDH19mut (X/Y)). (**a**) Brightfield images of CTRL and patient-derived iPSCs; (**b**) ALP staining of PCDH19mut iPSCs compared to CTRL iPSCs; (**c**) Analyses of genome stability of the generated PCDH19mut iPSCs (red bar graph) and CTRL iPSCs (blue bar graph); (**d**,**e**) Immunofluorescence assays in CTRL and PCDH19mut iPSCs for pluripotency markers OCT4 (**d**), SSEA4 (**d**), SOX2 (**e**) and TRA1-60 (**e**). Scale bar = 50 μm.

Three pluripotent iPSC clones were obtained from primary skin fibroblasts of an affected mosaic male individual (c.1352 C > T, p.Pro451Leu; [26]). Sanger sequencing confirmed the occurrence of the hemizygous PCDH19 variant after reprogramming (Figure S1a) and excluded the occurrence of any functionally relevant variant throughout the PCDH19-coding sequence in control iPSCs. Clones were selected based on the ESC-like morphology (i.e., rounded-shape colonies with defined edges) (Figure 1a), and pluripotency was confirmed by enzymatic assay using ALP (Figure 1b), positivity to pluripotency markers (Figure S1b), and differentiation into lineages belonging to the three germ layers (endoderm, mesoderm and ectoderm). 

**Figure 2:**
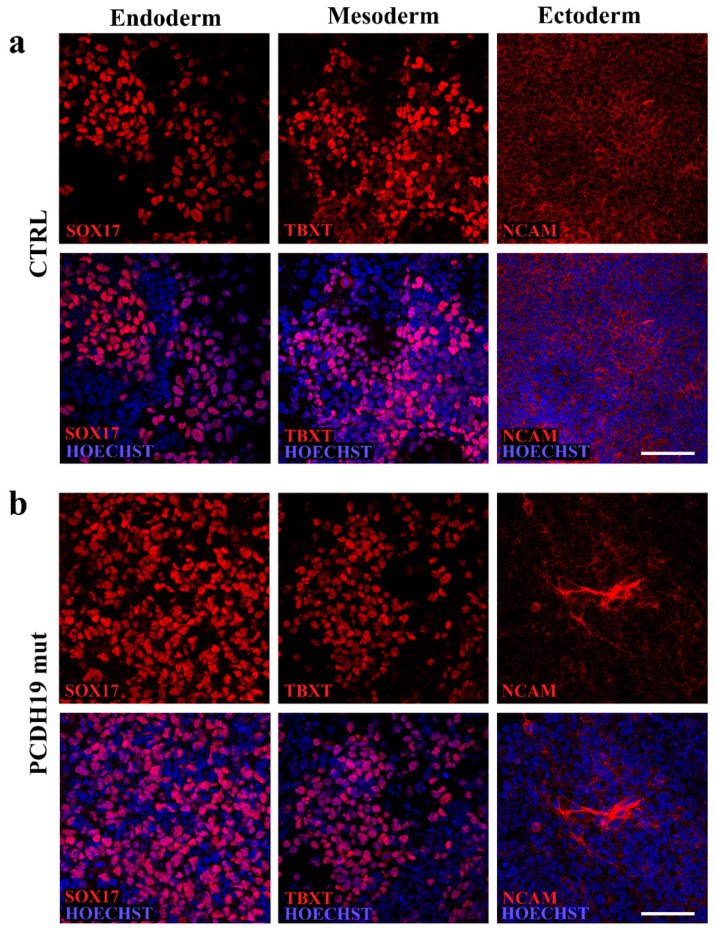
Characterization of the pluripotency ability of the selected iPSC clones. (**a**,**b**) Confocal micrographs of immunofluorescence analyses performed following differentiation into the three germ layers. The images show the expression of endoderm (SOX17), mesoderm (TBXT) and ectoderm (NCAM) markers in CTRL (**a**) and PCDH19mut (**b**) iPSCs. Scale bar = 50 μm.

Immunofluorescence assays for the pluripotency markers OCT4, SOX2, SSEA4 and TRA1-60 validated the full reprogramming of colonies (Figure 1d,e). The genomic integrity of the selected clones was verified by qPCR assay to exclude occurrence of the structural rearrangements which frequently occurred during reprogramming (Figure 1c). Finally, the ability of the lines to differentiate into cells belonging to the three germ layers was verified by assessing the protein expression of endoderm (SOX17), mesoderm (TBXT) and ectoderm (NCAM) markers by immunostaining (Figure 2a,b), confirming their pluripotency potential.

### 3.2. PCDH19-iPSCs Show Accelerated Differentiation In Vitro

**Figure 3:**
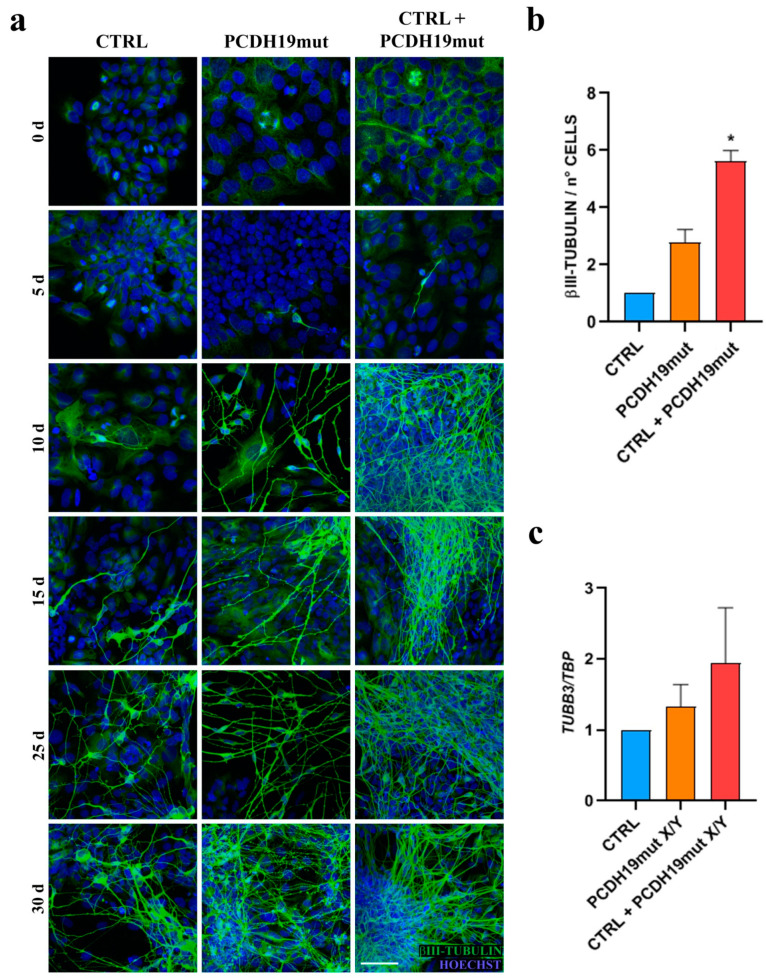
Neuronal differentiation of CTRL, PCDH19mut and mixed (CTRL + PCDH19mut) iPSC populations. (**a**) Micrographs of immunofluorescence assays for βIII-tubulin at different in vitro cortical neurogenesis timepoints, showing an accelerated differentiation of PCDH19mut and mixed cultures compared to CTRL. Scale bar = 50 μm; (**b**) Bar graph depicting the quantification of βIII-tubulin signal on the 30th day of differentiation in cortical neurons. Data are normalized to control and presented as the mean ± SEM, three biological replicates are indicated as *n* = 3. * *p* < 0.05, according to Kruskal–Wallis test; (**c**) Bar graph showing RT-qPCR analyses for *TUBB3* in PCDH19mut and mixed culture derived cortical neurons compared to CTRL cortical neurons. Data are presented as the mean ± SEM (normalized to control), *n* = 3. * *p* < 0.05, according to ordinary one-way ANOVA parametric test.

To investigate the role of PCDH19 during neurogenesis, iPSCs were differentiated into cortical neurons. The neuronal differentiation of the generated iPSC lines was assessed in vitro by immunofluorescence analysis. Since all iPSC clones obtained from the mosaic male patient were hemizygous for the PCDH19 variant, to recreate the mosaic conditions, CTRL and PCDH19mut iPSCs were cultured as a mixed culture (1:1 ratio). Immunofluorescence for the neuronal marker βIII-tubulin showed an increased number of neurons in the mixed culture compared to what was observed in individual line at each timepoint (Figure 3a). The quantification of βIII-tubulin-positive cells confirmed that, at the end of differentiation (T30), the mixed culture had a significantly increased signal compared to the parental cultures (Figure 3b). These findings were confirmed by independent experiments aiming to assess the expression levels of *TUBB3* mRNA by RT-qPCR assays, which consistently confirmed that there were increased expression levels in mixed cultures (although not statistically significant, Figure 3c). 

**Figure 4:**
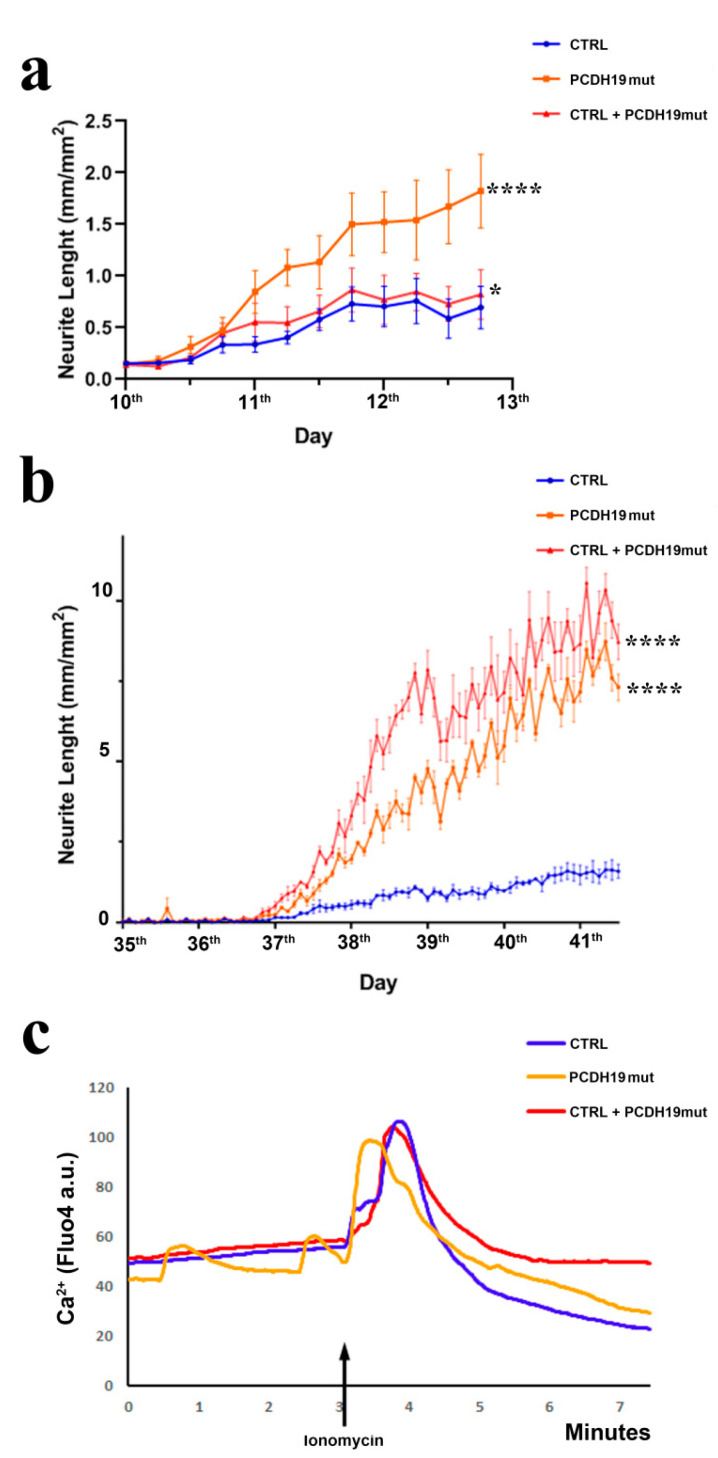
Morpho-functional phenotype of neurons derived from CTRL, PCDH19mut and mixed iPSCs. (**a**) Graph of live cell imaging assay of neurite length from the 10th to 13th day in CTRL, patient, and mixed iPSC-derived cortical neurons. Data are presented as mean ± SEM, *n* = 3. * *p* < 0.05, **** *p* < 0.0001, according to ordinary one-way ANOVA parametric test; (**b**) Neurite length analyses in CTRL, PCDH19mut and mixed cortical neurons showing neurite length from the 35th to 41st day. Data are presented as mean ± SEM, *n* = 3. **** *p* < 0.0001, according to ordinary one-way ANOVA parametric test; (**c**) Graphical representation of intracellular Ca^2+^ flux in CTRL, PCDH19mut and mixed cortical neurons, before (basal level), following stimulation with 20 μM ionomycin (at 3 min, as indicated by the black arrow) and following addition of EGTA to the medium (3′30′’); four biological replicates were performed (*n* = 4) and, for each replicate, 10 cells were analyzed.

Since an accelerated differentiation was observed in patient-derived neuronal cultures, morphological analyses of the obtained neurons were performed. To this end, we monitored neurite growth at the stage of neural rosettes’ formation, when the first differences among the neural cultures appear through live cell imaging analyses of neurite length from the 10th to 13th day. Neurites’ length, measured using the Incucyte System (Essen BioScience, Ann Arbor, MI, USA), documented that PCDH19mut and mixed neurons had significantly longer neurites when compared to CTRL neurons (**** *p* < 0.0001 and * *p* < 0.05, respectively) (Figure 4a). To accomplish a detailed analysis of neurites, we monitored the neurite growth at the end of neuronal differentiation, and quantified neurite length by performing a live cell imaging experiment from the 35th to 41st day, documenting that length was significantly increased in PCDH19mut and mixed neurons compared to CTRL neurons (Figure 4b).

To evaluate the neuronal activity, we measured intracellular calcium (Ca^2+^) levels, which are known to play a fundamental role in synaptic activity. In basal conditions, Ca^2+^ levels were lower in PCDH19mut cortical neurons and, following ionomycin, the maximal peak in intracellular calcium was decreased compared to CTRL. We also observed a spontaneous influx of Ca^2+^ in PCDH19mut neurons, even before ionomycin stimulation, thus representing a spontaneous Ca^2+^ influx. However, in the mixed culture, Ca^2+^ influx was similar to that of the CTRL culture (Figure 4c).

### 3.3. PCDH19-iPSCs Present an Altered Neural Rosette Organization

**Figure 5:**
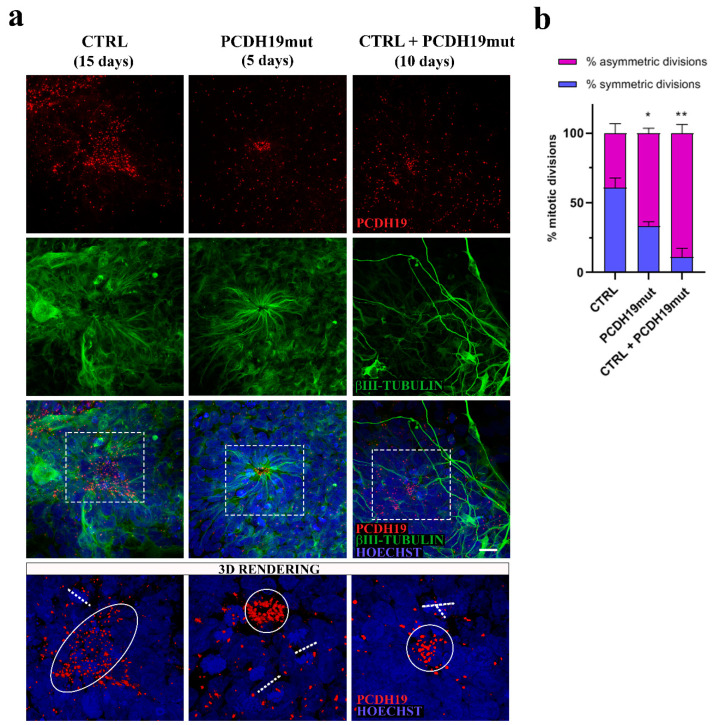
Analyses of dividing cells at the stage of neural rosettes. (**a**) Maximum intensity projections (MIP) of confocal micrographs of immunofluorescence assays for PCDH19 and βIII-tubulin showing the structure of neural rosettes derived from CTRL, PCDH19mut and mixed iPSCs. Scale bar = 20 μm. 3D rendering shows the type of division close to the center of the rosettes; (**b**) Quantification of symmetric and asymmetric cells in mitosis. Data are normalized to control and presented as mean ± SEM. Statistically significant difference is calculated with ordinary one-way ANOVA parametric test (* *p* < 0.05) (** *p* < 0,01), *n* = 3.

The rosette morphology and organization were analyzed by performing immunofluorescence assays using anti-PCDH19 and anti-βIII-tubulin antibodies. In CTRL cultures, the neural rosettes appeared around the 25th day of in vitro cortical neuronal differentiation, while they were distinguishable earlier (around the 10th day) in PCDH19mut cultures, showing a disorganized structure with reduced lumen. In the mixed CTRL + PCDH19mut culture, rosettes were not easily distinguishable, due to the high number of differentiated neuronal cells (Figure 5a).

To understand whether this defective organization was due to an enhanced predisposition of patient’s iPSCs toward neuronal differentiation, we performed RT-PCR analyses to quantify the levels of genes which were differently expressed during neurogenesis. Specifically, we investigated the expression levels of *NCAD*, as a marker of neuronal cells, and microtubule-associated protein 2 (*MAP2*) and *TUBB3* (encoding βIII-tubulin) as markers of mature neurons. Firstly, we evaluated *NCAD* levels to assess the predisposition of iPSCs to spontaneously differentiate them into neuronal cells. In patient’s iPSCs, cultured with and without CTRL iPSCs, the expression levels of this neuronal cadherin were increased compared to CTRL iPSCs, suggesting a predisposition towards neural differentiation (Figure S2). In support of this observation, the levels of *MAP2* and *TUBB3* mRNA were increased in PCDH19mut iPSCs compared to CTRL iPSCs, especially when cultured in mosaic condition, indicating that patient’s iPSCs expressed genes involved in neuronal differentiation, even without inducing neurogenesis, with defined culture media and specific growth factors (Figure S2). 

In vivo neurogenesis is tightly regulated by the fine equilibrium between the symmetric and asymmetric cell division of neural progenitors; at this stage of differentiation, the orientation of the mitotic spindle of dividing progenitor cells drives the cell fate of the dividing cells [31]. Specifically, cells that undergo symmetric division, with the mitotic spindle oriented perpendicular to the center of the rosette, will give rise to two daughter progenitor cells. On the contrary, cells undergoing asymmetric division will generate a neural progenitor and a cell that will undertake neuronal differentiation migrating away from the lumen of the rosette and resembling the migrating neurons that populate the layer of the cerebral cortex. For this reason, we analyzed and estimated the number of symmetric vs. asymmetric divisions close to the center of the neural rosettes and documented a significant increase in the percentage of asymmetric division in PCDH19mut and in mixed cultures compared to CTRL cultures (Figure 5b). 3D imaging allowed for appreciation of the asymmetric positioning of dividing cells in PCDH19mut and in mixed cultures, while those of CTRL cultures were dividing symmetrically. 

### 3.4. Mutated PCDH19 Affects Mitotic Spindle Formation

**Figure 6:**
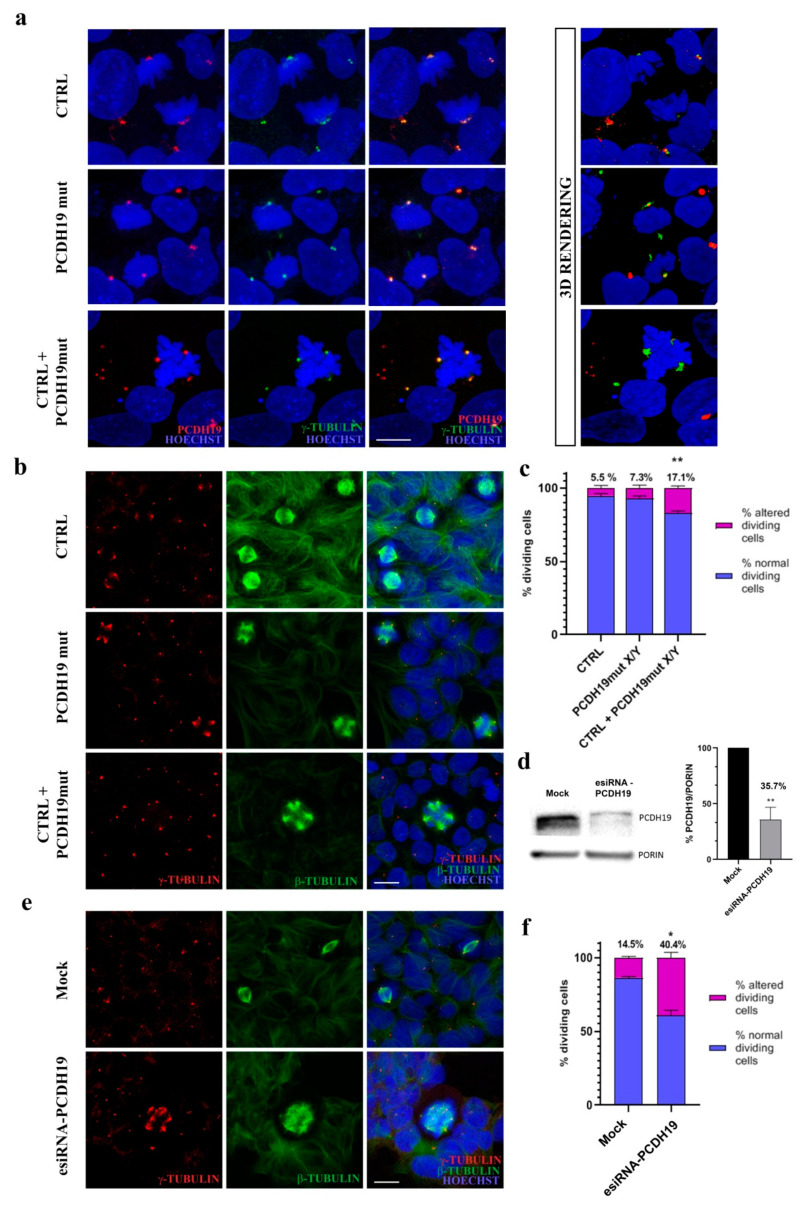
Analysis of the mitotic spindle in CTRL, PCDH19mut and mixed cell cultures and in PCDH19 silenced iPSCs. (**a**) Confocal micrographs and 3D reconstruction of immunofluorescence assays in CTRL, PCDH19mut and mixed iPSCs showing a comparable localization of PCDH19 and γ-tubulin. Scale bar = 5 μm; (**b**) Immunofluorescence analyses for γ-tubulin and β-tubulin showing the mitotic spindle structures in CTRL, PCDH19mut and mixed iPSCs. Scale bar = 5 μm; (**c**) Graph showing the quantification of anomalous metaphases in dividing cells. More than 1000 cells were analyzed for each sample. Data are presented as mean ± SEM (normalized to control), *n* = 3. ** *p* < 0.005, according to unpaired *t*-test; (**d**) Western blot assay and a bar graph quantification (of three independent experiments) display the amount of PCDH19 levels in CTRL (Mock) and PCDH19 silenced iPSCs (siRNA-PCDH19). Data are presented as mean ± SEM (normalized to control), *n* = 3. ** *p* < 0.005; (**e**) Images of immunofluorescence analyses for γ-tubulin and β-tubulin highlighting the mitotic spindle structures in PCDH19 silenced (siRNA-PCDH19) and CTRL iPSCs (Mock). Scale bar = 5 μm; (**f**) Graph depicting the quantification of altered metaphases in dividing cells in CTRL (Mock) and in silenced iPSCs (siRNA-PCDH19). More than 500 cells were analyzed for each sample. Data are normalized to control and presented as mean ± SEM. Statistically significant difference is calculated with unpaired *t*-test (* *p* < 0.05), *n* = 3.

Since the precocious differentiation of neurons is often related to alterations in the plane of cell division, we decided to investigate the effect that mutated PCDH19 has on the mitotic spindle. Importantly, we observed altered mitotic structures in patient’s and mixed iPSC populations. To further examine the impact of defective PCDH19 function on mitotic spindle organization, confocal analysis was directed to assessment of the possible cooperation between PCDH19 and a protein involved in the nucleation and polar orientation of microtubules. Therefore, we performed immunostaining assays for PCDH19 and γ-tubulin, which revealed the colocalization of these proteins (Figure 6a).

To evaluate possible perturbation on mitosis and microtubule spindle configuration in iPSCs derived from affected subject we performed immunostaining experiments using anti-γ- and anti-β-tubulin to reveal the mitotic spindle in dividing cells. We observed that proliferating PCDH19mut iPSCs cultured with CTRL iPSCs presented with multiple mitotic spindle structures during cell division (Figure 6b). Altered metaphases accounted for 17% of the total metaphases in the mixed culture, which was a significantly higher proportion compared to that observed inPCDH19mut iPSCs (7%) and CTRL iPSCs (4%) (Figure 6c). These results are consistent with the role of PCDH19 in regulating the plane of cell division, a process with great relevance during neurogenesis. 

### 3.5. Silencing of PCDH19 mRNA Alters Cell Division

To further understand the effect of PCDH19 loss of function, we performed Western blot assays directed to assess the reduction in the translated PCDH19 protein using silenced iPSCs. PCDH19 silencing was attained using a heterogeneous mixture of siRNA targeting the PCDH19 mRNA sequence (specifically, from exon 3 to 6) in CTRL iPSCs at a confluence of 500,000 cells. The levels of PCDH19, detected using the anti-PCDH19 antibody, resulted in 65.3% fewer silenced cells than were found in CTRL cells (Figure 6d). 

While documenting reduced levels of PCDH19 in immunofluorescence assays, we observed that the silenced cultures presented cells with altered mitotic spindle organization. To further investigate this aspect, the organization of the mitotic spindle was analyzed in dividing cells after PCDH19 silencing, which documented the presence of multipolar and aberrant mitotic spindle structures (Figure 6e). Data quantification showed that altered metaphases were significantly increased in silenced cells (esiRNA-PCDH19) when compared with those nucleofected with an empty vector (Mock) (Figure 6f). 

To evaluate if PCDH19 loss of function leads to altered metaphases with centrosome hyperamplification, we immunostained iPSCs with an anti-centriolin antibody. Confocal images show that PCDH19 was not only colocalized with γ-tubulin but also with centriolin in CTRL, patient’s and mixed iPSCs (Figure S3a). 

### 3.6. PCDH19mut iPSCs-Derived Brain Organoid Growth Is Decreased

**Figure 7:**
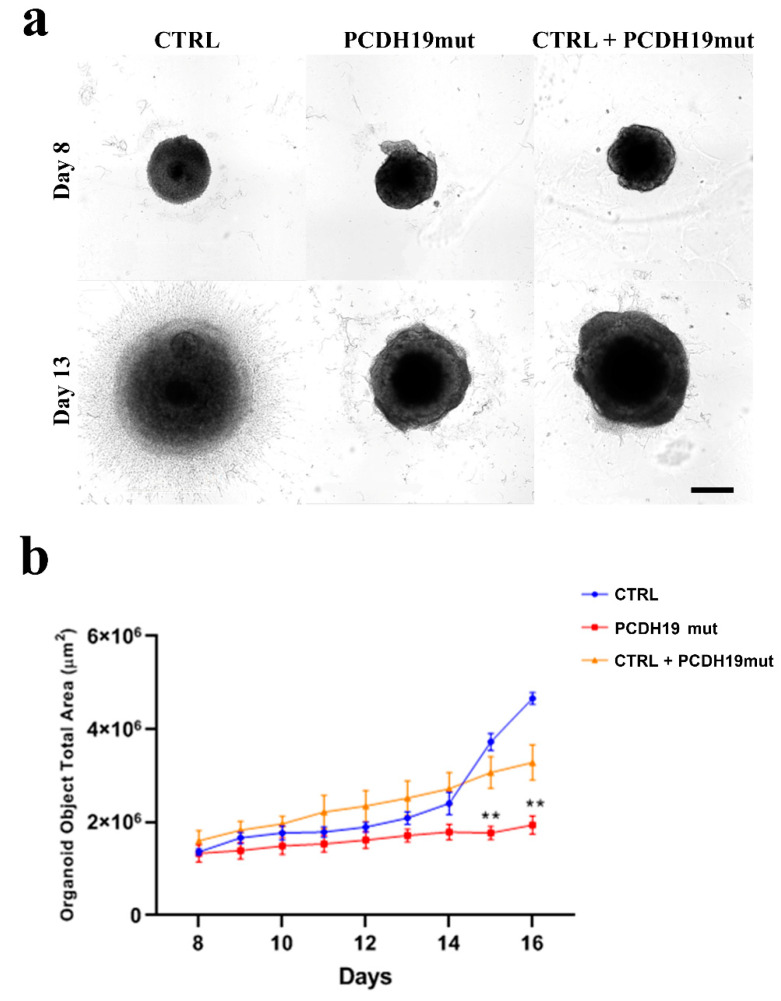
Brain organoids derived from CTRL, PCDH19mut and mixed iPSCs. (**a**) Brightfield images showing brain organoids at early stages of formation (from 8 to 13 days in vitro); (**b**) Graph representing the analysis of brain organoid size from the 8th day to the 16th day. Three organoids for each sample have been analyzed. Data are presented as mean ± SEM, *n* = 3. ** *p* < 0.005, according to ordinary one-way ANOVA parametric test.

To further model the PCDH19 neural phenotypes, we generated 3D cultures of brain organoids, and their size was assessed as a function of days in vitro (Figure 7a) At day 8, the total brain organoid area was indistinguishable among CTRL, PCDH19mut and mixed condition. However, by day 16, affected organoids (PCDH19mut and CTRL + PCDH19mut) were noticeably smaller than CTRL organoids (Figure 7b). 

## 4. Discussion

PCDH19 encodes a cell-surface-exposed adhesion molecule belonging to non-clustered protocadherins, expressed predominantly in the developing and adult brain. It is involved in calcium-dependent cell-to-cell adhesion, and studies in mice highlighted the role of PCDH19 in determining cell adhesion affinities during cortical development [21]. Moreover, PCDH19 is involved in regulating neurogenesis, since its loss of function leads to impaired neuronal migration and the accelerated development of cortical neurons [18,19,22,23]. Mutations or partial deletion of PCDH19 lead to an X-linked form of childhood clustering epilepsy, which is usually resistant to antiepileptic drugs [39,40], with early onset seizures, initially associated with fever. Unlike classical X-linked diseases, this disorder affects females and mosaic males. Mutations mainly affect exon 1, and include missense and nonsense changes, and small frameshift indels, affecting highly conserved amino acids, all in the extracellular domain of the protein [4]. PCDH19 mutations are thought to cause a loss of function of the protein [6,10]. With more than 175 mutations reported to date, PCDH19 is now clinically considered as the second major disease gene implicated in epilepsy, after *SCN1A* [4]. In addition to PCDH19, the defective function of other δ-PCDHs has been associated with neurological disease. For example, *PCDH10* mutations have been linked to autism [41,42], *PCDH12* mutations have been associated with schizophrenia [42] and microcephaly and seizures [43], and *PCDH17* mutations have been involved in the pathogenesis of schizophrenia [14]. These recent findings confirm a crucial role of non-clustered protocadherins in brain development.

PCDH19-CE is characterized by a variable phenotypic spectrum that ranges from benign focal epilepsy with normal intelligence to severe generalized/multifocal epilepsy, resembling Dravet syndrome. Some individuals with autism-like behavioral problems have also been reported [2,7,44,45,46]. Brain imaging with nuclear magnetic resonance is typically described as normal at onset of disease, but recent reports highlight the presence of acquired microcephaly [47], and structural lesions as cortical dysplasia, abnormal cortical sulcation, blurring of grey–white matter interface and clustering of dysplastic pyramidal neurons [19,39,48].

Since PCDH19-CE is poorly understood and an efficacious treatment is lacking, a better understanding of the pathophysiology of this disorder is required. A promising and informative in vitro model system is based on the modeling of cortical neuronal pathophysiology using patient-derived iPSCs. To achieve this goal, we reprogrammed patient’s fibroblasts into iPSCs. Multiple clones were characterized for their pluripotency and differentiated to cortical neurons. Our results demonstrate that iPSCs obtained from PCDH19 patients can undergo neurogenesis and are able to differentiate in cortical neurons, despite some alterations occurring, as was previously documented [22]. Different from what was observed in control iPSCs, whose neural rosettes are visible at day 15 of differentiation, the neural rosettes appear earlier in PCHD19mut iPSCs (day 5). In line with an accelerated neurogenesis in PCDH19mut cultures, neurites showed an increased length when compared with CTRL neurons. To further characterize the model, a mixed iPSC population culture was generated to obtain a mosaic condition to recapitulate the symptomatic mosaic males and heterozygous females and characterize the neuronal phenotype associated with cellular interference, the pathogenetic mechanism believed to underlie PCDH19-CE [6]. By comparing the growth and differentiation of individual and mixed control and patient’s iPSC cultures, we showed that accelerated differentiation occurs in PCDH19mut iPSCs. Interestingly, neuronal differentiation was significantly accelerated in mixed cultures. In line with these data, we observed that, during the last days of neural differentiation, the neurite length of the mixed cultures was significantly increased compared to CTRL cultures. Overall, these data unveil that increased neurogenesis occurs earlier in PCDH19mut cultures (with the appearance of precocious neural rosettes and an increased neurite length) and is even more accelerated in the mixed cultures (as observed for the *TUBB3* levels and neurite length). 

Before becoming adult neurons, stem cells undergo several maturation steps, in which they completely change their transcriptome. To unveil the molecular phenotype of our iPSC model system, we focused on the expression of a set of different genes that are expressed early during neurogenesis in vivo. NCAD is an adhesion glycoprotein expressed in neuroepithelium cells, with a critical role in neural progenitor cells’ fate, establishing whether they undergo symmetrical or asymmetrical division and, interestingly, cis-interacts with PCDH19 to reinforce cell adhesion [17,24]. Since NCAD is often used as a marker of neuronal lineage, the fact that its expression is increased in PCDH19mut and mixed iPSC cultures indicates that these cells have a spontaneous ability to differentiate toward neurons. This finding, in addition to the initial increase in neurite length and the early appearance of neural rosettes, suggests that patient’s iPSCs are more predisposed to neural differentiation. Consistently, mRNA analysis, considering MAP2 and TUBB3 as markers of mature neurons, revealed that PCDH19mut and mixed iPSCs were in line with an accelerated differentiation when compared to CTRL iPSCs. These results consistently support the role of PCDH19 loss of function in promoting neuronal differentiation, augmented in the mixed condition (thus supporting a non-cell autonomous mechanism for this phenotype).

To investigate the functionality of CTRL cortical neurons, we performed analyses of intracellular Ca^2+^ influx following ionomycin stimulation and observed that PCDH19mut cortical neurons show a spontaneous Ca^2+^ intracellular influx before ionomycin stimulation. These results suggest the increased excitability of PCDH19mut neurons and are in accordance with the increased excitability of hippocampal neurons, in which PCDH19 is downregulated [49]. Surprisingly, the Ca^2+^ imaging profile of mixed neurons is like that of CTRL neurons, although, following the addition of EGTA, the decrease in intracellular Ca^2+^ is delayed. This unexpected finding requires further study. 

Previous works used iPSCs as a model system to understand the localization of PCDH19 in stem cells and during cortical neurogenesis [50]. In iPSCs, PCDH19 is localized at one pole of the cell, and is possibly responsible for informing the position of one cell relative to the neighbouring cells. In addition, during cell division, PCDH19 is positioned at the two poles of the mitotic spindle, suggesting its involvement in the orientation of the spindle and the regulation of the type of cell division. Since in vivo neurogenesis is tightly regulated by the equilibrium between the symmetric and asymmetric cell division of neural progenitor cells (NPCs) in relation to their apical–basal polarity [32], and PCDH19 regulates this polarity [22], it is possible that PCDH19 mutations alter the equilibrium between symmetric vs. asymmetric division in a cell-autonomous manner, thus affecting neurogenesis. To focus on this, we investigated the orientation of mitoses in neural rosettes, which are structures developing during cortical neuronal differentiation by the re-organization of the cells and composed of NPCs positioned around a lumen which resembles the neural tube during in vivo neurogenesis. It is known that PCDH19 is highly expressed at the stage of neural rosettes and located at the centre of these structures, thus defining the proliferative zone [22,50]. We observed that the number of asymmetric divisions is significantly higher in PCDH19mut rosettes than in control and in mixed cultures (Figure 5b), suggesting that PCDH19 plays a role in informing the correct positioning of the mitotic spindle. PCDH19 dysfunction may, therefore, cause an imbalance between symmetric and asymmetric divisions in the proliferative zone of the neural rosette, leading to accelerated neural differentiation at the expense of the NPCs. According to this model, the authors of [51] recently showed that *Pcdh19* knock-down, through in utero intraventricular injection, results in a reduction in intermediate progenitor number in mice developing cortex. Moreover, an additional break-of-symmetry event, which is most likely what occurs in PCDH19mut and mixed cultures, leads to the appearance of the first neurite, thus expediting precocious neurogenesis.

Since PCDH19 is localized at the pole of the mitotic spindle in dividing cells [50] and its mutations alter the orientation of dividing cells during neurogenesis, we hypothesized that it interacts with proteins of the centrosome complex. To verify this hypothesis, we performed immunofluorescence assays for PCDH19, γ-tubulin, and centriolin in CTRL, PCDH19mut and mixed iPSC cultures. The colocalization of PCDH19 with both proteins of the centrosome was observed, and a significant alteration of mitotic spindle structures, with a consequent centrosome hyperamplification, was documented. To understand whether PCDH19 regulates the mitotic spindle formation, we also performed silencing experiments in CTRL iPSCs, documenting an increase in cells displaying multiple mitotic spindles, thus reinforcing the hypothesis that PCDH19 plays a role in controlling cell division. 

Since in vitro neurogenesis resulted accelerated in mixed iPSC cultures, we decided to assess the effects of PCDH19 on brain development using a 3D in vitro model. To accomplish this, we generated iPSCs-derived brain organoids and monitored their growth up to day 16th. On the 8th day, the PCDH19mut organoid area was indistinguishable from CTRL ones, but on the 13th day, they appeared to be smaller than CTRL organoids. In particular, an analyses of the total area show that, from the 14th day, PCDH19mut organoid growth was significantly decreased. The fact that the WT cells show an initially slow proliferation growth, followed by a dramatic increase in the organoid area after day 14, would reflect an initial time when the cells need to reorganize their transcriptional machinery under the influence of a specific growth factor before undergoing neural 3D differentiation, while the PCDH19mut and CTRL + PCDH19mut cells have an innate predisposition towards neural differentiation. These results suggest that brain organoids from PCDH19-CE patients are reduced in size, modeling the increased neurogenesis observed in 2D cultures. Further analyses are needed to better characterize the cellular structure of the brain organoids.

**Figure 8:**
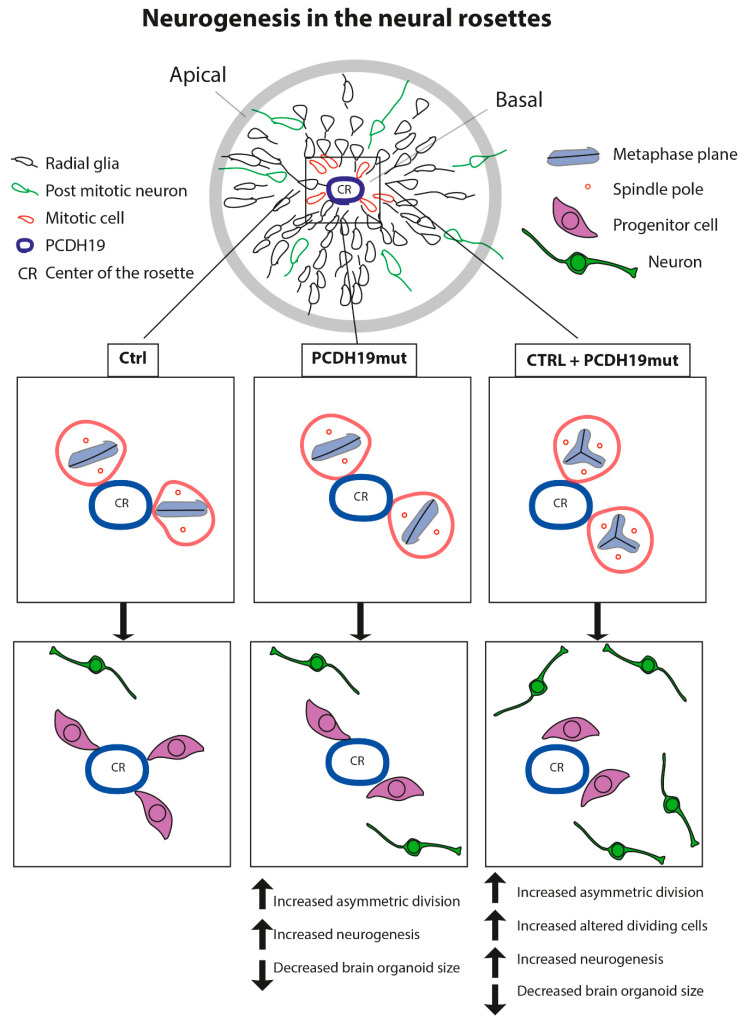
Schematic drawing depicting the model we used to explain the effect of PCDH19 on dividing cells at neural rosette stage in CTRL, PCDH19mut and mixed iPSC cultures. Scheme of a neural rosette showing mitotic cells (in red) close to the center of the rosette (CR) (corresponding to basal zone), post-mitotic neurons (in green) which are migrating away from the lumen and the radial glia (or progenitor cells) toward the apical zone. Boxes show the orientation of metaphase plan in CTRL, PCDH19mut and mixed (CTRL+PCDH19mut) dividing cells. In CTRL rosettes, two cells are dividing: one in a symmetric and the other in an asymmetric way, generating two neural progenitor cells and one progenitor and one post mitotic neuron, respectively (in total, three progenitors and one neuron). In PCDH19mut rosettes, two cells are undergoing asymmetric division, and each generates one progenitor and one post-mitotic neuron (in total, two progenitor cells and two neurons). Mutated PCDH19 is responsible for a “cell autonomous” effect, which induces an increase in asymmetric divisions, resulting in increased neurogenesis and decreased brain organoids’ size. In mixed iPSCs-derived rosettes, two cells divide, forming a multiple mitotic structure and resulting in increased neurogenesis (in total, two progenitor cells and four neurons). In addition to the cell-autonomous effect in PCDH19mut cell, the coexistence of mutated PCDH19 and wild type PCDH19 results in a “non-cell-autonomous” effect that induces the formation of altered dividing cells, which led to an exacerbation of the increased neurogenesis phenotype.

Together, our findings provide evidence that PCDH19 regulates neurogenesis by controlling the mitotic spindle organization and its mutations lead to accelerated in vitro differentiation, which is in line with previous studies [22]. The accelerated neurogenesis is further evinced by the increased mRNA levels of genes relevant for neural differentiation at the iPSC stage (which suggests an increased susceptibility of PCDH19mut cells to differentiate), through the precocious appearance of rosettes. Of note, the increased percentage of asymmetric cell division of NPCs and altered mitotic cells in mixed iPSCs likely represent the molecular events explaining the observed increased neural differentiation rate leading to the observed decreased brain organoid size (Figure 8). In line with this hypothesis, mice with increased numbers of centrosomes present neural stem cell disorientation, decreased numbers of progenitor cells and premature neuronal differentiation [52]. In accordance with our findings, cortical dysplasia is a recurrent finding in PCDH19-CE [19,39,48], and acquired microcephaly has also been reported [48]. In the future, it would be important to deepen the morpho-functional phenotype of the brain organoids, derived from PCDH19mut iPSCs using isogenic cell lines, to more precisely investigate the processes related to neuronal differentiation and function [36,53,54,55]. These studies represent a required step for the development of effective pharmacological treatments. The generation and characterization of model systems is a required step for the development of effective pharmacological treatments. Among in vitro disease models, iPSC-derived neurons appear to be well-suited for use in drug-screening strategies aiming to develop targeted therapeutic approaches, and represent an informative experimental tool to understand pathogenesis. Here, we characterized the neuronal phenotype of PCDH19-CE in terms of cell morphology, intracellular Ca^2+^ flux, cell division and organoid formation/organization, and the collected data suggest that drugs acting on microtubule polymerization dynamics might be considered as slowing down the cell cycle in order to counteract premature differentiation of neural progenitor cells.

## Figures and Tables

**Table 1:** Primers used for RT-qPCR experiments. The annealing temperature for all primers is 62 °C.

Gene	Forward	Reverse
*TBP*	5′-CCGAAACGCCGAATATAATCC-3′	5′-AAATCAGTGCCGTGGTTCGT-3′
*OCT4*	5′-AGCGAACCAGTATCGAGAAC-3′	5′-TTACAGAACCACACTCGGAC-3′
*SOX2*	5′-AGCTACAGCATGATGCAGGA-3′	5′-GGTCATGGAGTTGTACTGCA-3′
*TUBB3*	5′-GCCGCTACCTGACGGTGGC-3′	5′-AGCGAACCAGTATCGAGAAC-3′
*NCAD*	5′-GCCACCTACAAAGGCAGAAG-3′	5′-CCGAGATGGGGTTGATAATG-3′
*MAP2*	5′-CTACAGCTGAGCCTTCAGACC-3′	5′-TCTTCCAGGCTGGCAACAAG-3′

## Supplementary Materials

The following are available online at https://www.mdpi.com/article/10.3390/jcm10132754/s1, Figure S1: Characterization of PCDH19mut iPSCs. (a) Sanger sequencing in PCDH19mut iPSCs. Electropherogram shows the pathogenic mutations (c.1352 C>T) in iPSC clones derived from a mosaic male patient. The arrow indicates the position of the mutation; (b) Relative expression levels of *OCT4* and *SOX2* mRNA in PCDH19mut iPSCs compared to CTRL iPSCs. Data are presented as the mean ± SEM (normalized to control), *n* = 4. One-way ANOVA test was used to perform statistical analysis of the obtained data. Figure S2: qRT-qPCR results in CTRL, patient’s and mixed iPSCs of several markers expressed during neurogenesis. Bar graphs show mRNA levels of *NCAD*, *MAP2* and *TUBB3*. Data are normalized to control and presented as the mean ± SEM, n = 3. * *p* < 0.05, according to ordinary one-way ANOVA parametric test. Figure S3: (a) Confocal micrographs of immunofluorescence images for PCDH19 (red) and centriolin (green) in CTRL, PCDH19mut and mixed iPSCs. Scale bar = 10 μm; (b) confocal micrographs showing PCDH19 (red) and centriolin (green) colocalization respectively during normal and altered mitoses in CTRL and silenced iPSCs. Scale bar = 5 μm; (c) Confocal micrographs of immunofluorescence for γ-TUBULIN (red) and centriolin (green) showing normal and altered mitoses respectively in CTRL and silenced iPSCs. Scale bar = 5 μm.
