# Supplementary material for: Dissecting the Role of PCDH19 in Clustering Epilepsy by Exploiting Patient-Specific Models of Neurogenesis
Source: J Clin Med. 2021 Jun 23;10(13):2754. doi: 10.3390/jcm10132754 (PMC8268119; doi:10.3390/jcm10132754)
Supplement: Supplementary file 1 [file jcm-10-02754-s001.zip › jcm-1240324-supplementary.pdf]

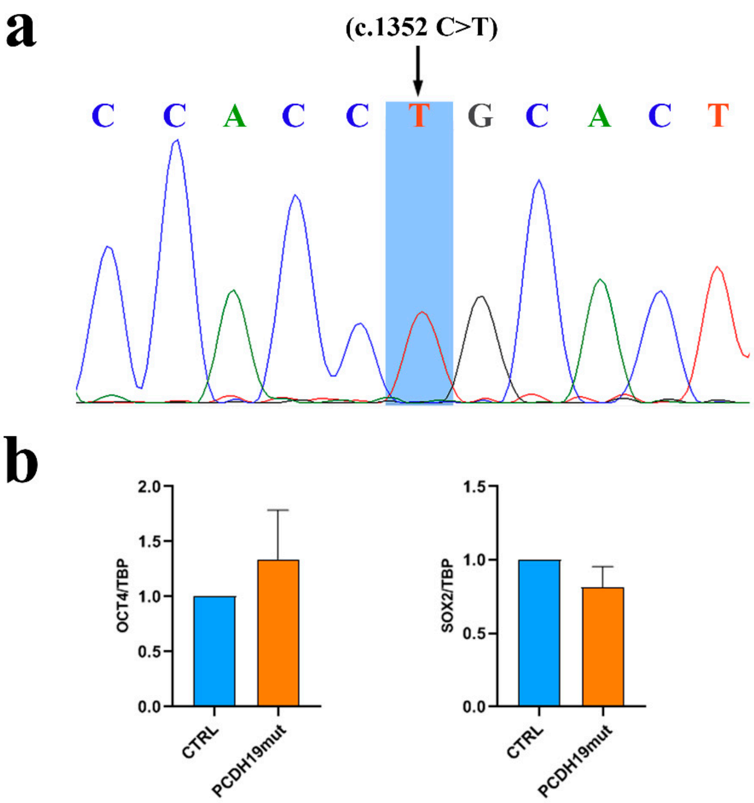

Figure S1. Characterization of PCDH19mut iPSCs. (**a**) Sanger sequencing in PCDH19mut iPSCs. Electropherogram shows the pathogenic mutations (c.1352 C>T) in iPSC clones derived from a mosaic male patient. The arrow indicates the position of the mutation; (**b**) Relative expression levels of OCT4 and SOX2 mRNA in PCDH19mut iPSCs compared to CTRL iPSCs. Data are presented as the mean ± SEM (normalized to control), $n = 4$. One-way ANOVA test was used to perform statistical analysis of the obtained data.

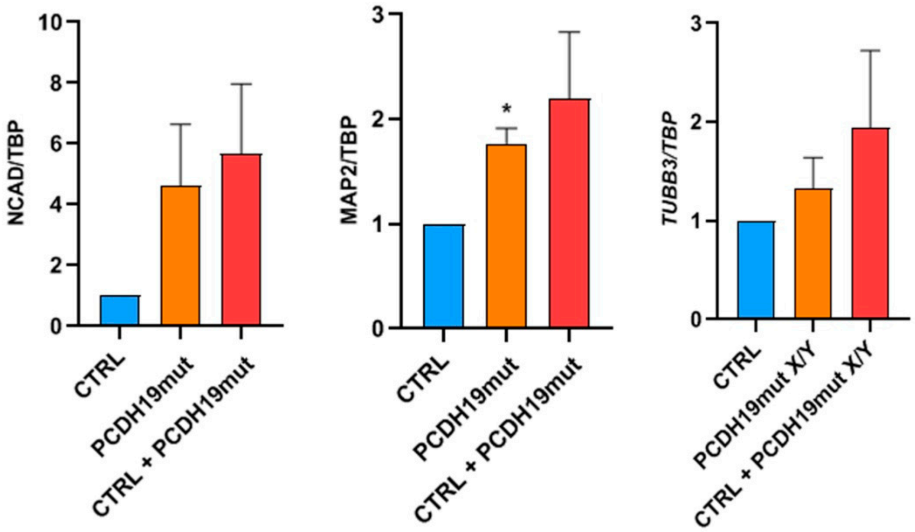

Figure S2. qRT-qPCR results in CTRL, patient's and mixed iPSCs of several markers expressed during neurogenesis. Bar graphs show mRNA levels of NCAD, MAP2 and TUBB3. Data are normalized to control and presented as the mean ± SEM, $n = 3$. * $p < 0.05$, according to ordinary one-way ANOVA parametric test.

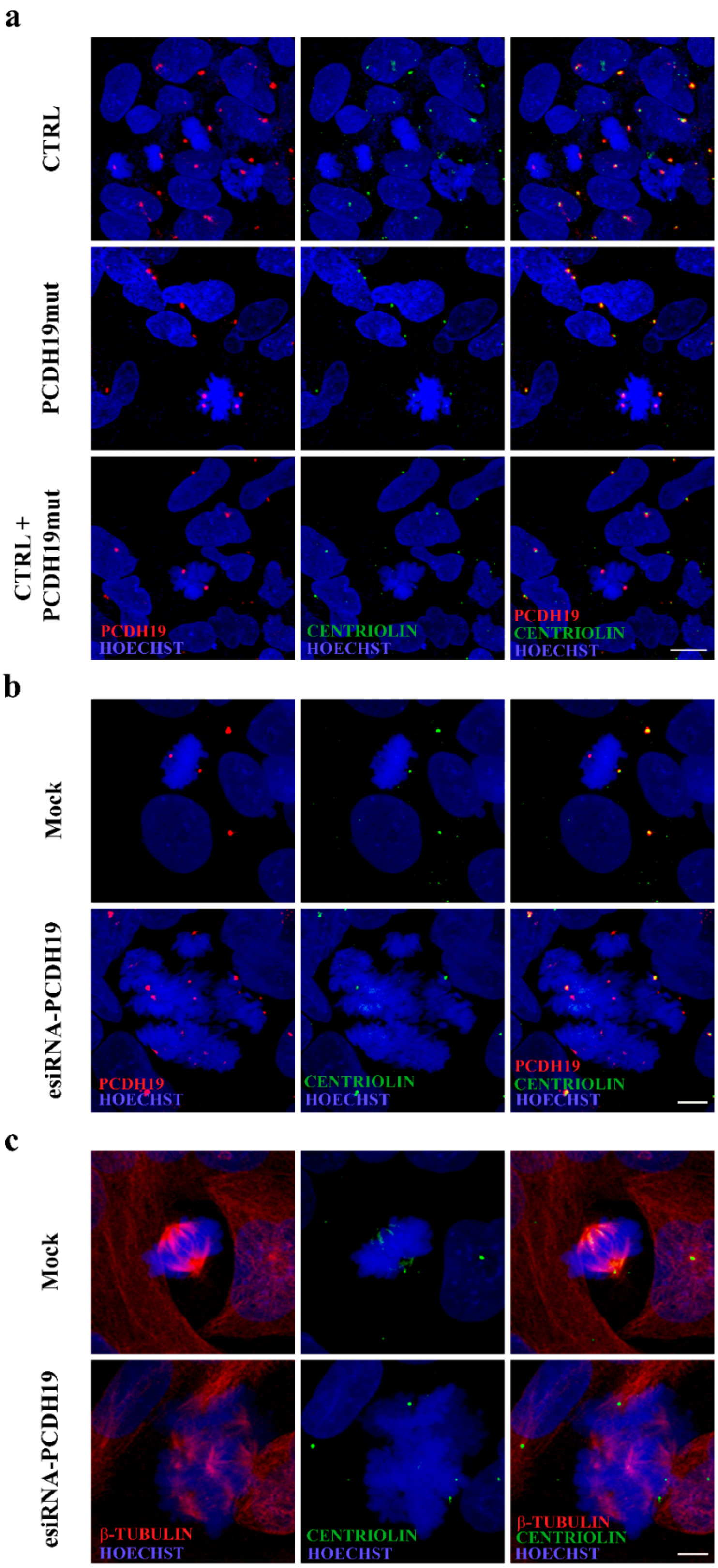

Figure S3. (**a**) Confocal micrographs of immunofluorescence images for PCDH19 (red) and CENTRIOLIN centriolin (green) in CTRL, PCDH19mut and mixed iPSCs. Scale bar =1 0μm; (**b**) confocal micrographs showing PCDH19 (red) and CENTRIOLIN centriolin (green) colocalization respectively during normal and altered mitoses in CTRL and silenced iPSCs. Scale bar= 5 μm; (**c**) Confocal micrographs of immunofluorescence for γ-TUBULIN (red) and CENTRIOLIN centriolin (green) showing normal and altered mitoses respectively in CTRL and silenced iPSCs. Scale bar= 5 μm.
